# Investigating emotion regulation and social information processing as mechanisms linking adverse childhood experiences with psychosocial functioning in young swiss adults: the FACE epidemiological accelerated cohort study

**DOI:** 10.1186/s40359-022-00798-5

**Published:** 2022-04-11

**Authors:** Jeannette Brodbeck, Salome I. R. Bötschi, Neela Vetsch, Thomas Berger, Stefanie J. Schmidt, Simon Marmet

**Affiliations:** 1 School of Social Work, University of Applied Sciences and Arts Northwestern Switzerland, Riggenbachstrasse 16, 4600 Olten, Switzerland; 2 Department of Clinical Psychology and Psychotherapy, University of Bern, Fabrikstrasse 8, 3012 Bern, Switzerland; 3 Department of Clinical Child and Adolescent Psychology, University of Bern, Fabrikstrasse 8, 3012 Bern, Switzerland

**Keywords:** Adverse childhood experiences, Psychosocial functioning, Emotion regulation, Rejection sensitivity, Interpretation bias, Service use, Young adulthood, Social support

## Abstract

**Background:**

Adverse childhood experiences increase the risk for psychological disorders and lower psychosocial functioning across the lifespan. However, less is known about the processes through which ACE are linked to multiple negative outcomes. The aim of the FACE epidemiological study is to investigate emotion regulation (emotional reactivity, perseverative thinking and self-efficacy for managing emotions) and social information processing (rejection sensitivity, interpretation biases and social understanding) as potential mechanisms linking adverse childhood experiences and psychosocial functioning in a large population sample of young adults. It is embedded in a larger project that also includes an ecological momentary assessment of emotion regulation and social information processing and informs the development and evaluation of an online self-help intervention for young adults with a history of ACE.

**Methods:**

The study plans to recruit 5000 young adults aged 18 to 21 from the German-speaking Swiss population. Addresses are provided by Swiss Federal Statistical Office and participants are invited by mail to complete a self-report online survey. If the targeted sample size will not be reached, a second additional sample will be recruited via educational facilities such as universities or teacher training colleges or military training schools. Three follow-ups are planned after 1 year, 2 years and 3 years, resulting in ages 18–24 being covered. The main exposure variable is self-reported adverse childhood experiences before the age of 18, measured at the baseline. Primary outcomes are psychosocial functioning across the study period. Secondary outcomes are social information processing, emotion regulation and health care service use. Statistical analyses include a range of latent variable models to identify patterns of adverse childhood experiences and patterns and trajectories of psychosocial adaptation.

**Discussion:**

The results will contribute to the understanding of the underlying mechanisms that link ACE with psychosocial functioning which is crucial for an improved insight into risk and resilience processes and for tailoring interventions. Furthermore, the identification of factors that facilitate or hinder service use among young adults with ACE informs healthcare policies and the provision of appropriate healthcare services.

*Trial registration number*: NCT05122988. The study was reviewed and authorized by the ethical committee of Northwestern and Central Switzerland (BASEC number 2021-01204).

## Background

Childhood experiences affect psychosocial functioning and mental health across the life course for better or worse. Positive family experiences and social support promote high well-being and represent a buffer against stressors. Still, one out of three children growing up worldwide experiences adverse childhood experiences (ACE) such as emotional, physical and sexual abuse, emotional and physical neglect, exposure to intimate partner violence or other household dysfunctions [1]. A recent review of meta-analyses found worldwide self-reported prevalence rates of 36.3% for emotional abuse, 22.6% for physical abuse, 12.7% for sexual abuse (18% for girls and 7.6% for boys), 18.4% for emotional neglect and 16.3% for physical neglect [1].

ACE are a well-established transdiagnostic risk factor for impaired psychosocial functioning and various mental and physical health conditions throughout life. This includes depression, anxiety, post-traumatic stress disorder (PTSD), suicidal behaviour, obesity, cardiovascular diseases, and other medical conditions [2–4]. However, less is known about the processes through which ACE are linked to multiple negative outcomes. A better understanding of the underlying processes is crucial for an improved insight into risk and resilience processes across the lifespan.

In the context of ACE, emerging adulthood, covering the ages of 18 to about 25 years, is a crucial stage in life that offers a window of opportunity for recovery, but also holds a risk for the consolidation of problems, deterioration of trajectories or the onset of destructive outcomes. Young adults start to take control of their own lives and are better able to distance themselves from an adverse family environment [5]. Self-regulation capacity and executive functioning increase and facilitate planning ahead and the consideration of alternatives [6]. Multiple transitions occur, including the shift from economic dependence to independence, moving out of the parents’ home, forming new relationships, and entering new stages of professional or educational life [7]. These transitions offer turning points and opportunities for redirecting maladaptive trajectories into healthier paths, but also pose challenges to the psychosocial adaptation of young adults [5, 8, 9]. Insights into such trajectories and their underlying processes is critical to inform interventions targeting risk factors for the negative effects of ACE in emerging adulthood.

Processes linking ACE with psychosocial functioning may depend on the subtype of ACE. ACE represent distinct experiences such as physical, emotional or sexual abuse, neglect, or household dysfunctions such as parental substance use or psychopathology which can have different consequences on physical and psychological health and psychosocial adaptation. However, different definitions of ACE and the fact that ACE often co-occur [2] hinder the integration of results. The analysis of the effects of ACE traditionally employs a single risk approach, examining only one subtype of ACE and ignoring the high co-occurrence of ACE, or use a cumulative risk approach, summarizing different forms of ACE and not accounting for the fact that ACE represent distinct experiences [10]. A more recent development addressing this issue is the Dimensional Model of Adversity and Psychopathology (DMAP) by McLaughlin, Sheridan and colleagues [10–13] which conceptualises ACE within underlying dimensions of adversities. These dimensions are the level of threat associated with abuse and level of deprivation associated with a lack of cognitive inputs or social stimulation as well as neglect. These dimensions have a differential impact on cognitive and emotional development. The exposure to threat in early life has been linked with higher perceptual sensitivity and attention biases towards negative emotional stimuli and patterns of information processing that prioritise threat-related information, higher emotional reactivity, and poor emotion regulation. Deprivation has been associated with impaired cognitive learning, i.e., in the domain of language and executive functioning.

### Mechanisms linking ACE and psychosocial functioning

The present study focuses on two important mechanisms also posited in the DMAP that are known to be affected by ACE and are risk factors for psychosocial adaptation and psychological disorders later in life: emotion regulation and social information processing. Deficits in *emotion regulation* has been established as a direct risk factor for psychological disorders and as a mechanism that mediates the relation between ACE, psychosocial functioning and different mental health problems [9, 14–19]. Higher emotional reactivity, rumination, and less adaptive emotion regulation strategies, as well as ineffective responses to distress, link ACE with internalising and externalising psychopathology in adolescence [16, 20]. *Social information processing,* including rejection sensitivity, social interpretation biases and social understanding have been found to link ACE to a lower psychosocial functioning. ACE deprive children and adolescents of socio-emotional support within the family and represent pathogenic relational experiences in an environment which is supposed to give care. Attachment theories point out that this can result in an insecure or disorganised attachment style, dysfunctional internal working models of the self, of others and of how relationships work which are risk factors for later psychological disorders [21, 22]. Internal working models provide an inadequate basis for appropriate social information processing and hinder engagement in good social relationships.

Rejection sensitivity, i.e., the tendency to anxiously expect, perceive and overreact to social rejection, attention and interpretation biases, as well as social understanding in tandem with emotion regulation, play an important role in social functioning, affect tendencies to approach or withdraw from others and reactions of interaction partners [23, 24]. Rejection sensitivity has been confirmed as a mediator for the associations between emotional abuse and depressive symptoms in adulthood [23–26]. Studies investigating social understanding, hostile attribution bias and attention biases in individuals with a history of ACE have yielded inconsistent results [27]. Negative interpretation biases were found to mediate the association between rejection sensitivity and depressive symptoms [28].

Theory and empirical findings suggest that maladaptive internal working models, interpretation biases and rejection sensitivity can lead to less favourable social behaviour that hinders the building up of good relationships and seeking social support [22]. Maltreated children are at risk for social withdrawal, aggressive behaviour or both [29, 30] and a history of ACE is linked to lower social motivation, less social support and higher social isolation in adults [31, 32]. Lower social support and loneliness have been established as a mechanism linking ACE to adolescent or adult well-being and psychopathology [32–37].

Apart from social support, professional help is an important factor that can mitigate the consequences of ACE on psychosocial functioning in young adulthood. However, many individuals who meet the criteria for psychological disorders do not seek or receive treatment [38]. There is a lack of knowledge on factors and processes that determine whether someone seeks, receives, and accepts social and professional support, especially among individuals with a history of ACE. Therefore, identifying the facilitating and hindering factors for receiving treatment and service use among young adults with ACE can help to improve access to appropriate interventions and promote the use of mental healthcare services.

### The present study

The present study aims to overcome several shortcomings of previous studies and theoretical models: Firstly, while most theoretical models and empirical studies have focused either on emotion regulation or social information processing, the FACE study combines these two strands of research and investigates the longitudinal interplay and relative strength of emotion regulation and social information processing as mediators for the link of ACE and psychosocial functioning. This is relevant, as the intensity of emotions and emotion regulation may affect different social information processing tasks. Lemerise and Arsenio’s Integrated Model of Emotion Processes and Cognition in Social Information Processing [39] posits that a database of memories, social schemas, social knowledge and acquired rules as well as the intensity of emotions, emotional processing and emotion regulation affect all stages of social information processing, including the perception and encoding of cues, their interpretation, the clarifications of goals, and finally the behavioural response. These factors may affect good social relationships and seeking and receiving social support.

Secondly, while many previous studies ignore the subtypes of ACE as potential moderators for differential health outcomes and employ a single risk or a cumulative risk approach, the FACE epidemiological study uses comprehensive measures for assessing distinct patterns of ACE. This may provide a better understanding of more complex risk environments [40]. Such patterns could then be related to patterns of psychosocial adaptation, such as vulnerable, resilient or average psychosocial functioning. Complementary to this categorical approach, we will investigate a dimensional approach to vulnerability and resilience which defines resilience as “doing better than expected” compared to individuals with similar levels of adversity [41, 42].

Thirdly, the FACE epidemiological study uses a longitudinal design complemented by an ecological momentary assessment in a large population-based sample which can disentangle the short- and long-term temporal interplay of emotion regulation and social information processing. It thus provides a better understanding of the mechanisms underlying the association between ACE and psychosocial functioning.

**Fig. 1:**
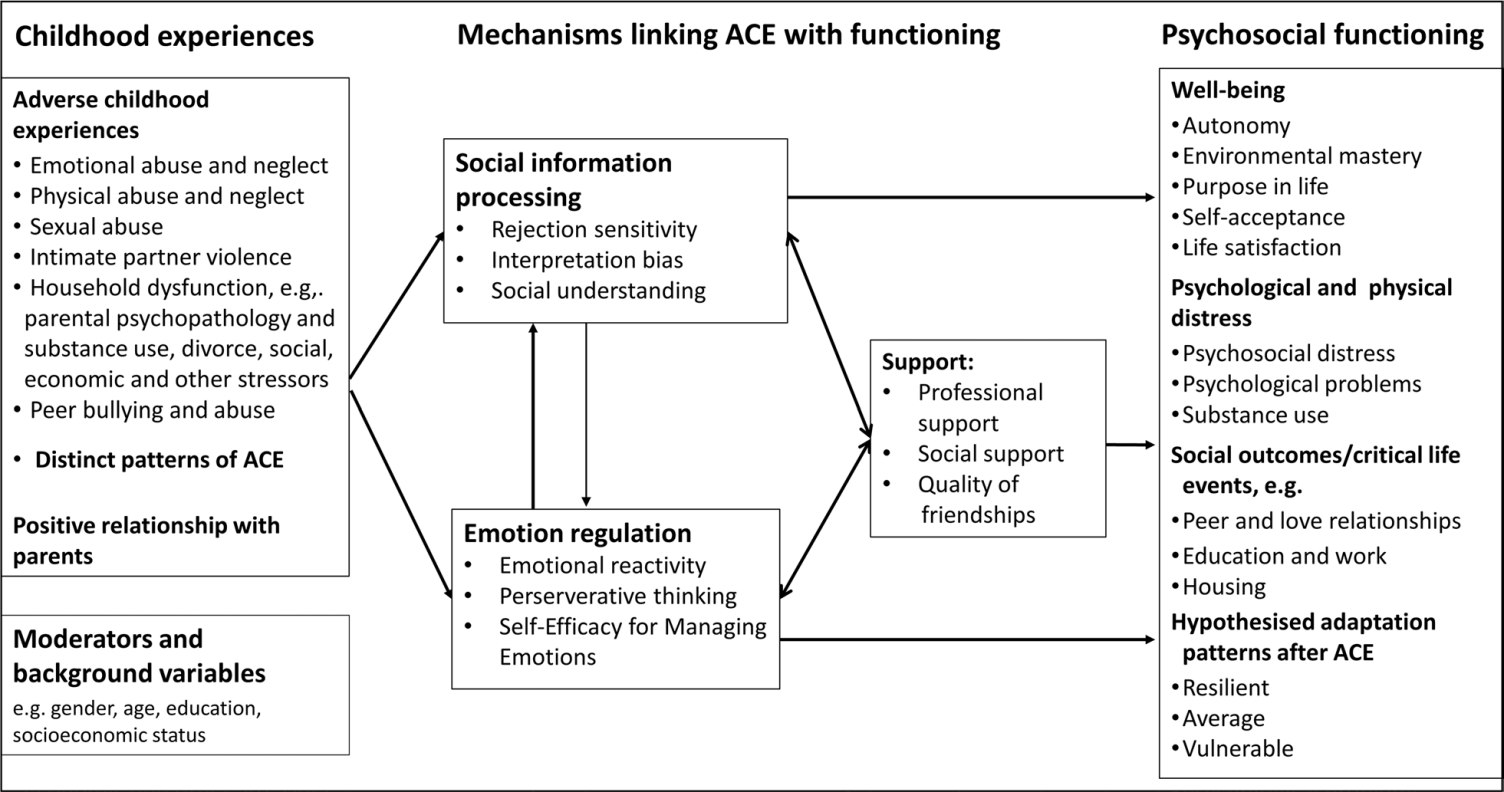
Overview of the Integrated Model of Emotion Regulation and Social Information Processing in the Aftermath of ACE

In sum, the major aim of this study is a comprehensive analysis of processes and mediators that link ACE and psychosocial functioning in emerging adults in the framework of the Integrated Model of Emotion Regulation and Social Information Processing in the Aftermath of ACE (see Fig. 1). Furthermore, we aim to identify factors that facilitate or hinder the use of mental healthcare services and finding social support among young adults with history of ACE.

## Methods

### Objectives

The primary objective of the study is to examine the impact of self-reported ACE on psychosocial functioning and to investigate the longitudinal interplay and relative strength of emotion regulation and social information processing, as processes and mediators linking ACE with psychosocial functioning in young adults in the framework of the Integrated Model of Emotion Regulation and Social Information Processing in the Aftermath of ACE. A multivariate approach will identify distinct adaptation patterns of psychosocial functioning, such as chronic maladaptive, intermittent maladaptive, resilient and recovery trajectories [43, 44]. The main hypotheses are that ACE are associated with a higher risk for lower psychosocial functioning in young adulthood and that this association is mediated by (a) deficits in emotion regulation, i.e., higher emotional reactivity, more perseverative thinking and lower self-efficacy for managing emotions, and (b) social information processing, i.e., higher rejection sensitivity, higher levels of interpretation biases and lower social understanding. Referring to the DMAP, we expect that emotion regulation shows a stronger indirect effect for the association between the threat dimension of ACE and psychosocial functioning than for the association between the deprivation dimension of ACE and psychosocial functioning. Furthermore, the FACE epidemiological study aims to identify factors that facilitate or hinder the use of mental healthcare services among young adults with history of ACE.

Secondary objectives are to analyse the effects of emotion regulation and social information processing on the quality of friendships and social support; to explore the longitudinal associations of emotion regulation and social information processes on facets of psychosocial functioning and distress and to investigate the longitudinal associations between emotion regulation and social information processing. Additional explorative analyses investigate differential effects of distinct patterns of ACE on emotion regulation and social information processing as well as psychosocial functioning. Figure 1 gives an overview of the assessed variables and the hypothesised associations between them.

### Study design

The FACE epidemiological study consists of an accelerated cohort design with four waves, which allows to cover the development between the ages of 18–24 years within a three-year investigation. Participants fill out online self-report questionnaires at baseline (2021; w1) and in three yearly follow-ups (2022: w2, 2023: w3, 2024: w3).

**Fig. 2:**
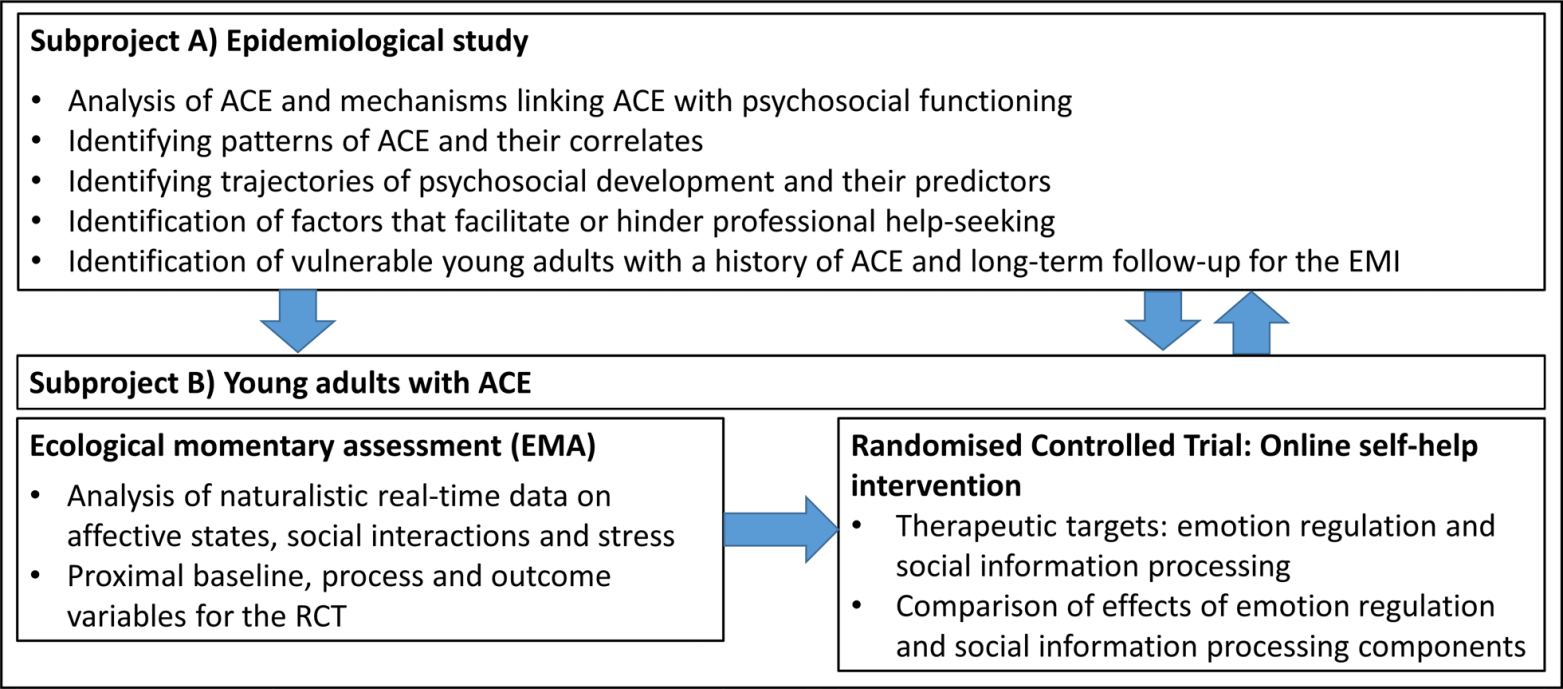
Overview of the subprojects and their role in the FACE project

The FACE epidemiological study (subproject A in Fig. 2) is embedded in a larger project that also includes the development and evaluation of an online self-help intervention for young adults with ACE (subproject B). The self-help intervention targets self- and emotion regulation and social skills and information processing and will be evaluated in a randomised clinical trial (RCT). Subproject B also includes an ecological momentary assessment study to provide real-life data for testing the efficacy of the intervention and further investigating the interplay of ACE, emotion regulation and social information processing. The epidemiological study informs the development of the intervention and identifies young adults with ACE who will be invited to take part. The later waves of the epidemiological study will serve as a long-term follow up for the intervention and allow a comparison to young adults with ACE who were not willing to take part in the intervention. A detailed study protocol for subproject B will be published in due course.

### Participants

The target population are 5000 young adults from the general population. Inclusion criteria are an age between 18 and 21 at baseline and the provision of an informed consent. Exclusion criteria are insufficient mastery of the German language, a cognitive or physical inability to take part in the online questionnaire reported by a relative, for example reported by a relative, or the inability to follow the studies procedures, e.g., due to not having internet access.

Addresses of 15,000 young adults are provided by the Swiss Federal Office of Statistics. Participants are drawn at random from all individuals living in a private household in German-Speaking municipalities that did not already participate recently in another survey. We will send an invitation letter with the study information to all young adults by postal mail. The invitation letter also lists psychosocial services and helplines and a small giveaway in form of summer flower seeds. Participants have also the chance to take part in a participation-associated lottery with monetary winnings. For the first wave, participants can win 200 vouchers for a Swiss Supermarket chain for 50 CHF (approximately 55 US Dollars). For each of the next waves, they can win 75 vouchers for 50 CHF. Such incentives have been shown to increase response rates [45, 46]. The invitation letter contains an identification number and a link to the study webpage. Using their unique 6-digit identification number, participants can give their written informed consent electronically on REDCap [47, 48] by signing with their mouse or touch screen before starting to fill out the online questionnaires.

We expect a response rate of 30%. If the targeted sample size will not be reached, a second sample will be recruited via educational facilities such as universities, teacher training colleges or military training schools. A link to the study website with the full study information and the informed consent will either be posted on university webpages and other billboards or forwarded via intermediary student organisations. Invitations for follow-ups will be sent to the e-mail address provided by participants in the baseline questionnaire. For the follow-ups, two reminders per e-mail are planned for participants not replying to the invitation.

### Measures

Measures used in the study are presented in Table 1. Apart from adverse childhood experiences and some socio-demographic variables, all measures will be assessed at baseline and at the three follow-up waves. Wherever possible, we used validated translations. Measures that were not available in German were translated by multiple team members into German. Differences in translations were resolved by discussion. The translations were also double checked for comprehensibility by university students who are close to the age range of the target population.

**Table 1:** Overview of the measures

Childhood experiences (only at t1), main exposure variable	
Adverse childhood experiences	- Child Maltreatment. Childhood Trauma Questionnaire, 5 subscales (physical and emotional neglect and abuse, sexual abuse), 5 items each [49, 50]. To assess the timing and chronicity of ACE, all participants reaching the subscale threshold for slight to moderate maltreatment were additionally asked in which years of their life this subtype of maltreatment occurred. - Witnessing violence to other family members (5 items); verbal and physical abuse by peers (6 items). Items were adapted from the German version of the Maltreatment and Abuse Chronology of Exposure’(MACE) scale [51, 52]. Additional item were added for the timing and chronicity per scale.
Family context and relationships	- Family Context before age 18, 3 items. Adapted from the C-SURF study [53]. - Satisfaction with family relationships before age 18: mother, father, and siblings. Adapted from the C-SURF study [53]. - Psychiatric problems of parents and siblings, Adapted from the C-SURF study [53].
**Psychosocial functioning**	
Well-being	- Ryff’s Psychological Well-Being Scale, 42 items, 7 for each dimension (autonomy, environmental mastery, personal growth, positive relations with others, purpose in life, and self-acceptance) [54, 55], German translation adapted from Staudinger (1990) [56].
Life satisfaction	- Diener Satisfaction with life scale [57], 5 items. German version [58]. - Additional questions about satisfaction with different types of relationships: to friends, to romantic partner, to parents and to family/relatives.
Work and social adjustment	- Work and Social Adjustment Scale, 5 items [59].
Psychosocial and somatic problems and distress	- Brief Symptom Inventory (BSI-18) assessing symptoms of somatisation, depression and anxiety [60]. - Externalising Problem Screener, 10 items [61]. - Psychosocial distress in 13 different areas of life, for example school/work, sleep, romantic relationship, physical health, financial situation, adapted from Brodbeck (2007) [62]. - Diagnosis of COVID and Long COVID, worsening of psychological distress due to COVID-19. - RIWA Critical Life Events, 21 items, adapted from Brodbeck (2007) [62]. - Suicidal ideation and attempts, 2 items from the Suicidal Behaviours Questionnaire-Revised [63, 64]. On this page, a phone number and webpage in case of need for help is indicated.
Substance use	- Frequency of use for tobacco, alcohol, cannabis, party drugs, cocaine/heroin, other drugs, and pharmaceutical drugs for non-medical use. - For substance used: age at first use and the 4-item Addiction Screener [65].

Secondary outcomes and potential mediators for the association between ACE and primary outcomes	
**Emotion regulation**	
Emotional reactivity	- Emotion Reactivity Scale, 21 items [66].
Perseverative thinking	- Perseverative Thinking Questionnaire, 15 items [67].
Self-efficacy for managing emotions	- PROMIS Short Form v1.0—Self-Efficacy for Managing Emotions, 7 items [68].
**Social information processing**	
Rejection sensitivity	- Rejection Sensitivity Questionnaire, 9 scenarios with 2 questions each [69, 70].
Interpretation bias	- WSAP-hostility scale for hostile interpretation bias, 16 items with 2 questions each [71]. - Interpretation bias index for PTSD, 13 items [72].
Social understanding	- Tromsoe Social Intelligence Test: Social awareness & social information processing, 14 items [73].
**Social and professional support**	
Social support	- Social Support Questionnaire, short Version, (F-SozU K-14), 14 items [74] plus one item on informational support.
Professional support and service use	- Use of child and youth counselling services, ambulant and residential psychological and psychiatric treatment as well as child protection services, 5 items (partially only at t1).

### Statistical analysis

Significance level will be two-sided α = 0.05. Due to the large sample size, effect sizes will be prioritised over the significance level for the interpretation of the results. Cohort data will be analysed with a range of observed and latent variable models using Mplus that include person- and variable-centred approaches. Distinct patterns of ACE will be identified using a Latent Class Analysis based on the subtype and frequency of childhood experiences, for example patterns combining subtypes of child maltreatment, household dysfunctions or bullying by peers. Psychosocial functioning as primary outcome will be analysed in a variable- and a person-centered approach. For the variable-centered approach, a composite measure of psychosocial functioning will be computed using Factor Analyses including bifactor models. For the person-centered analyses, a Latent Profile Analysis is used to identify psychosocial adaption patterns, for example an average group, a resilient group, a moderately vulnerable group, and a highly vulnerable group.

The main hypotheses for the associations between childhood experiences, emotion regulation, social information processing and psychosocial functioning will be analysed using structural equation models with subtypes, dimensions, or patterns of childhood experiences as predictor and moderator variables and psychosocial functioning as outcome variable. (Moderated) mediation hypotheses will be tested using structural equation modelling with the simultaneous inclusion of dimensions of emotion regulation and social information processing variables as mediators linking childhood experiences with psychosocial functioning.

The trajectories of psychosocial adaptation, emotion regulation strategies and social information processing will be investigated with Growth- and Growth-Mixture models. For the longitudinal trajectories of psychosocial functioning across emerging adulthood, we expect to find a chronically dysfunctional group, a recovering group and a resistant trajectory group over time using Latent Growth Mixture Models. The effect of the use of mental health care services after baseline will be analysed with a Latent Transition Analysis where service use will be entered as predictor and the transition between the subgroups as outcome variable.

Logistic regression analyses will be used to analyse predictors of service use and enrolment to the FACE intervention (as binary variables). Predictor variables are simultaneously entered in the model and include ACE, age, gender, socio-economic status and psychosocial functioning.

Where an influence of the COVID-19 pandemic is to be expected, we will adjust for the impact of the crisis on individuals to ensure that trends are not created by the consequences of the COVID-19 epidemic or conduct sensitivity analysis to gauge the effect of the COVID-19 crisis. For example, there may be a general improvement of mental health in case the pandemic is ending between two waves of our study, and it will be important to account for that when considering trends across the four waves.

*Justification of sample size:* The sample size of the epidemiological study is determined with consideration of the number of participants needed in the FACE intervention study, i.e., 350 participants. This sample size allows the detection of small effect sizes for the interaction between time (pre, post, follow-up) and the two treatment condition SSIP vs SER, mediation analyses with parallel mediators in a structural equation modelling framework and, a multi-group model for each treatment arm, and an accurate estimates of the regression coefficients, the variance components and the standard errors for multilevel analyses of the EMA data [75, 76], details will be reported elsewhere]. For the FACE *epidemiological study*, the sample size of 5000 at baseline is based on the expectations that 25% (n = 1250) of the participants report ACE and that a third of them (n = 375) give an informed consent for the intervention study and start the FACE self-help programme.

*Handling of missing data:* An attrition of 40% is expected in the fourth wave of the epidemiological study, resulting in a sample of about 3000 participants at the fourth wave. We will conduct missingness analyses which identify patterns and predictors of missingness and determine whether missing data are completely at random, at random or non-ignorable. If missingness at random is supported, we will use Multiple Imputations in Mplus [77]. Sensitivity analyses will compare the results of different ways of imputing missing data including complete case analyses and if appropriate full information maximum likelihood.

*Data storage:* The electronic consent, the questionnaires and the case report forms are programmed in REDCap. Time, table, data field and altered value, and the person are recorded (audit trail). A role concept with personal passwords (members of the study team, i.e., principal investigator, research associates and assistants) regulates permission for each user to use the system and database as he/she requires. For each data extract, quality checks are carried out, the data is adjusted and made securely available in a coded form. The final data set will be available to interested researchers after the end of data collection in accordance with the funding agency’s open data policy.

## Discussion

Adverse childhood experiences have a high prevalence, cause considerable personal suffering and are a well-established transdiagnostic risk factor for various mental and physical health conditions throughout life. However, less is known about the processes through which ACE are linked to multiple negative outcomes. A better understanding of the underlying processes is crucial for an improved insight into risk and resilience processes and for tailoring interventions.

The main aims of the FACE epidemiological study are to investigate the associations between ACE and psychological functioning and to investigate to which degree these associations are mediated by deficits in emotion regulation, social information processing and lower social support. It is the first study that links two strands of theory and research, i.e., emotion regulation and social information processing in the context of ACE, and analyses the short- and long-term longitudinal interplay of ACE, emotion regulation, social information processing, social support and psychosocial functioning. The results will substantiate the Integrative Model of Emotion Regulation and Social Information Processing in the Aftermath of ACE. The longitudinal design with a large sample size will provide an opportunity to provide new insights into causal mechanisms linking ACE to psychosocial functioning and to test moderating effects of several other factors such as gender or socioeconomic factors. With its large sample size, the study will allow to identify different patterns of ACE, providing new insights into how ACE co-occur and how these patterns are related to distinct trajectories of psychosocial functioning.

Furthermore, the FACE epidemiological study provides data on self-reported ACE as well as service use among young adults to inform healthcare policies and the provision of appropriate healthcare services. It prospectively identifies factors that facilitate or hinder service use in general and the use of the FACE self-help programme specifically. The FACE project examines which young adults are especially receptive to an intervention –and the age at which they would be most receptive—as well as seeking to identify the developmental stage (regarding life transitions) at which the intervention is most effective. The combined data of both subprojects provide the empirical basis to advance theory-driven models of enrolment and engagement with m-health interventions.

This study has several limitations. All data from participants are self-reported and thus subject to several types of bias, notably recall bias (especially events in early childhood), social desirability bias, non-response bias, or hesitation to answer sensitive questions. Data collection for the baseline assessment will take place during the ongoing COVID-19 pandemic, and the COVID-19 situation continues to have an impact on participants economic, social, and psychological situation, and may impact trends in psychosocial functioning. We will closely follow the further development of the pandemic and adjust for its effect where appropriate. Selective attrition between study wave is a potential limitation, as those with a more negative development of psychosocial functioning may reply less often to follow up. However, multiple imputation procedures can account for this.

In conclusion, the FACE project is the first study that links two strands of research and analyses the longitudinal interplay of emotion regulation and social information processing in young adults with and without ACE as: (a) long-term associations in a large sample of the epidemiological study; as (b) ecological momentary assessments; and (c) as targets of the m-health intervention. It takes patterns and dimensions of ACE as moderators into account and includes a comprehensive assessment of psychosocial outcome measures as dimensions and patterns of vulnerability and resilience. It will allow to investigate causal mechanisms linking ACE to psychosocial functioning, with a focus on social information processing, emotion regulation, and social and professional support.

## Data Availability

The datasets used and/or analysed during the current study will be available from the corresponding author on reasonable request.

## Abbreviation

ACE: Adverse childhood experiences
